# Crystal Structures, Vibrational Spectra, and Fungicidal Activity of 1,5-Diaryl-3-oxypyrazoles

**DOI:** 10.3390/molecules19011302

**Published:** 2014-01-21

**Authors:** Yi Li, Yuanyuan Liu, Yihuang Xiong, Xiaohui Xiong

**Affiliations:** 1 College of Food Science and Light Industry, Nanjing University of Technology, Nanjing 211816, China; E-Mail: liynj2012@njut.edu.cn; 2 Department of Chemical and Pharmaceutical Engineering, Southeast University ChengXian College, Nanjing 210088, China; 3 Department of Materials Science and Engineering, the Pennsylvania State University, University Park, Pennsylvania, PA 16802, USA; E-Mail: yyx5048@psu.edu

**Keywords:** 1,5-diaryl-3-oxypyrazoles, crystal structure, fungicidal activity, structure-activity relationship

## Abstract

The aryloxypyrazole structure is present in a number of bioactive molecules. Four 1,5-diaryl-3-oxypyrazoles containing benzoyl (**I**), thiazolidinethione (**II** and **III**) or *per*-*O*-acetylated glucopyranosyl (**IV**) moieties were characterized by single-crystal X-ray diffraction. Compounds **I** and **II** crystallize in a triclinic *P*-*1* system, whereas **III** and **IV** crystallize in an orthorhombic *Pbca* and a monoclinic *P2_1_* space groups, respectively. The dihedral angles between the two benzene rings of the pyrazole are 61.33° (**I**), 62.87° (**II**), 57.09° (**III**) and 70.25° (**IV**). The structures were stabilized by classical intra- (C-H···S for **II** and **III**, C-H···O for **IV**) and intermolecular (C-H···O for **I** and **IV**) H-bonds, as well as intermolecular C-H···π stacking interactions. The theoretical FTIR results showed good agreement with the experimental data. Compounds **IV**, **II** and **III** showed moderate fungicidal activity against *Sclerotinia sclerotiorum* and *Gibberella zeae*. The structure-activity relationships were discussed.

## 1. Introduction

Since the discovery of the fungicide pyraclostrobin by BASF scientists [1,2,3], aryloxypyrazoles have attracted enormous attention due to their diverse bioactivities in fungicide [4,5], insecticide [6], and herbicide [7]. We have also devoted considerable effort to develop this series of fungicide, and found several 1,5-diaryl-3-oxypyrazoles containing alkyloxyacetate, heterocycle, glucopyranosyl or benzoyl moieties with fungicidal activity [8,9,10,11,12]. However, their detailed structural properties and structure-activity relationship have not been reported.

**Figure 1:**
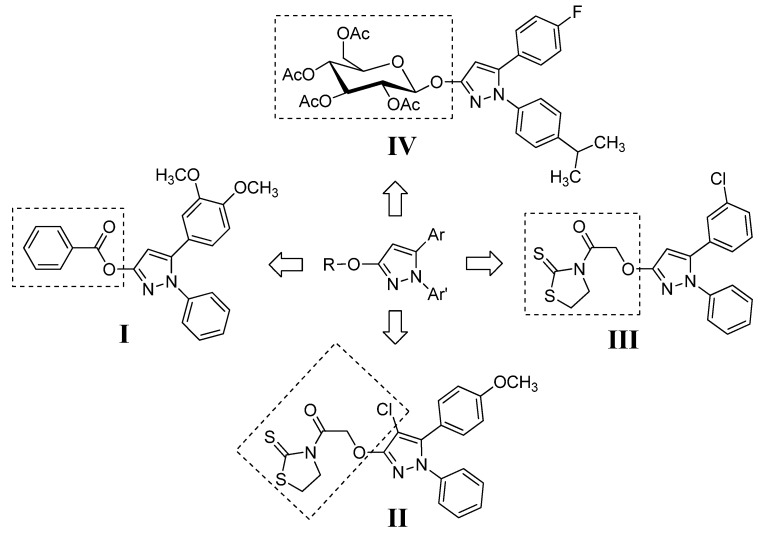
The four 1,5-diaryl-3-oxypyrazoles **I**, **II**, **III**, and **IV**.

In this paper, we report the crystal structures and FTIR spectra of four 1,5-diaryl-3-oxypyrazoles bearing benzoyl (**I**), thiazolidinethione (**II** and **III**) or *per*-*O*-acetylated glucopyranosyl (**IV**) moieties (Figure 1). Meanwhile, their *in vitro* fungicidal activity against *Sclerotinia sclerotiorum* and *Gibberella zeae* has been investigated, and their structure-activity relationshipe were also discussed.

## 2. Results and Discussion

### 2.1. Structural Description

The detailed crystal and structure refinement data of **I**–**IV** are listed in Table 1. Compounds **I** and **II** crystallize in a triclinic *p*-1, whereas **III** and **IV** crystallize in an orthorhombic *Pbca* and a monoclinic *P2_1_* space groups, respectively. Selected bond lengths and angles/torsion angles for **I**–**IV** are given in Table 2. The bond lengths of N1-C4 (1.417(7) Å) and N3-C17 (1.412(6) Å) in **II** and **III** are longer than normal N-C amide bond (1.325-1.352 Å) [13]. The C7-Cl bond length in **II** is 1.732(6) Å, which is similar to the aryl-Cl value of 1.730(7) Å (C11-Cl) in **III**. The C16-F bond length in **IV** is 1.380(7) Å, representing a typical alkyl-F bond, and the value is similar to those (1.371(3) Å) reported for other related derivatives [14]. The bond angle of C9-C10-C11 in **I** is 104.2(3)°, whereas the corresponding bond angles in **II**–**IV** are 106.3(5)° (C6-C7-C8), 104.9(4)° (C13-C14-C15), and 104.4(5)° (C10-C11-C12), respectively. These values are similar to the typical angle values of five-membered rings (108.0°).

**Table 1:** Crystal data and structure refinements for **I**, **II**, **III** and **IV**.

	I	II	III	IV
CCDC	906894	819778	918082	925316
Empirical formula	C_24_H_20_N_2_O_4_	C_21_H_18_ClN_3_O_3_S_2_	C_20_H_16_ClN_3_O_2_S_2_	C_32_H_35_FN_2_O_10_
Formula weight	400.42	459.95	429.93	626.62
Temperature	293(2)	293(2)	293(2)	293(2)
Wavelength (Å)	0.71073	0.71073	0.71073	0.71073
Crystal system	Triclinic	Triclinic	Orthorhombic	Monoclinic
Space group	P-1	P-1	Pbca	P2_1_
*a* (Å)	9.3040(19)	6.0180(12)	13.698(3)	11.838(2)
*b* (Å)	10.507(2)	8.2370(16)	7.6260(15)	9.4500(19)
*c* (Å)	12.075(2)	21.600(4)	38.908(8)	14.514(3)
*α* (°)	68.70(3)	87.08(3)	90.00	90.00
*β* (°)	81.40(3)	87.46(3)	90.00	100.41(3)
*γ* (°)	67.99(3)	82.30(3)	90.00	90.00
Volume (Å^3^)	1,019.5(4)	1,058.9(4)	4,064.4(14)	1,596.9(6)
*Z*	2	2	8	2
Calculated density (Mg/m^3^)	1.304	1.443	1.405	1.303
Absorption coefficient (mm^−1^)	0.090	0.406	0.414	0.101
*F*(000)	420	476	1,776	660
Crystal size (mm)	0.10 × 0.20 × 0.30	0.05 × 0.05 × 0.10	0.10 × 0.20 × 0.30	0.10 × 0.20 × 0.30
*θ* range for data collection(°)	1.81–25.36	1.89–25.28	1.05–25.28	1.43–25.25
Index ranges	0 ≤ h ≤ 11 −11 ≤ k ≤ 12 −14 ≤ l ≤ 14	0 ≤ h ≤ 7 −9 ≤ k ≤ 9 −25 ≤ l ≤ 25	0 ≤ h ≤ 16 0 ≤ k ≤ 9 0 ≤ l ≤ 46	−14 ≤ h ≤ 13 0 ≤ k ≤ 11 0 ≤ l ≤ 17
Reflections collected	4002	4239	3688	3091
Independent reflections [*R*(int)]	3750 [*R*(int) = 0.045]	3843 [*R*(int) = 0.061]	3688 [*R*(int) = 0.034]	3091 [*R*(int) = 0.052]
Max. and min. transmission	0.9911 and 0.9736	0.9800 and 0.9605	0.9597 and 0.8857	0.9900 and 0.9704
Refinement method on *F*^2^	Full-matrix least-squares	Full-matrix least-squares	Full-matrix least-squares	Full-matrix least-squares
Data/restraints/parameters	3750/0/271	3843/0/272	3688/0/253	3091/7/388
Goodness-of-fit on *F*^2^	1.005	1.002	1.032	1.004
Final *R* indices [*I* > 2*σ*(*I*)]; *R*_1_, *wR*_2_	*R*_1_ = 0.0688 *wR*_2_ = 0.1508	*R*_1_ = 0.0854 *wR*_2_ = 0.1592	*R*_1_ = 0.0740 *wR*_2_ = 0.1616	*R*_1_ = 0.0656 *wR*_2_ = 0.1567
*R*_1_, *wR*_2_ (all data)	*R*_1_ = 0.1452 *wR*_2_ = 0.1835	*R*_1_ = 0.1766 *wR*_2_ = 0.1897	*R*_1_ = 0.1359 *wR*_2_ = 0.1922	*R*_1_ = 0.1000 *wR*_2_ = 0.1798
Largest diff. peak and hole (e·Å^−3^)	0.149 and −0.169	0.451 and −0.266	0.325 and −0.358	0.226 and −0.577

**Table 2:** Selected bond lengths (Å), bond angles/torsion angles (°) for **I**, **II**, **III**, and **IV**.

Comp.	Bond lengths (Å)	X-ray	Bond angles/Torsion angles (°)	X-ray
**I**	C11-O3	1.391(4)	C9-C10-C11	104.2(3)
	N1-N2	1.374(3)	N2-N1-C11-O3	173.5(3)
	C5-C9	1.472(4)	N1-N2-C9-C5	−172.8(3)
	O3-C18	1.360(4)	C1-O1-C8-C7	5.0(5)
			C2-O2-C3-C4	−2.7(5)
**II**	C6-O2	1.326(8)	C6-C7-C8	106.3(5)
	N1-C4	1.417(7)	N3-N2-C6-O2	−179.7(6)
	C7-Cl	1.732(6)	N2-N3-C8-C9	−176.1(6)
	N2-N3	1.357(6)	C15-O3-C12-C11	1.1(11)
	C8-C9	1.479(8)	C4-N1-C3-S2	−1.0(10)
	O2-C5	1.414(7)		
**III**	C15-O1	1.351(5)	C13-C14-C15	104.9(4)
	N3-C17	1.412(6)	N1-N2-C15-O1	178.1(4)
	C11-Cl	1.730(7)	N2-N1-C13-C7	177.8(4)
	N1-N2	1.365(5)	C17-N3-C18-S1	9.4(7)
	C7-C13	1.477(7)		
	O1-C16	1.424(5)		
**IV**	C10-O1	1.392(6)	C10-C11-C12	104.4(5)
	C16-F	1.380(7)	N2-N1-C10-O1	177.4(6)
	N1-N2	1.388(5)	N1-N2-C12-C13	−176.3(6)
	C12-C13	1.499(7)	O1-C19-O2-C20	−173.8(4)
	O1-C19	1.404(6)	O2-C19-C23-O10	−174.2(4)
			C21-C20-C26-O4	−61.0(6)
			C19-O1-C10-C11	160.7(6)

X-ray diffraction studies indicated that the dihedral angles between the *C*-linked benzene ring A and *N*-linked benzene ring B of **I**, **II**, **III**, and **IV** are 61.33°, 62.87°, 57.09°, and 70.25°, respectively. Rings A, B and benzoyloxy ring C in **I** are twisted 50.95°, 40.62°, and 64.18°, respectively, from the plane of the pyrazole ring. The dihedral angle between rings A and C is 32.20°, whereas the corresponding angle between rings B and C is 47.99°. The N2-N1-C11-O3 and N1-N2-C9-C5 torsion angles are 173.5(3)° and −172.8(3)°, whereas the corresponding angles in **II**, **III**, and **IV** are −179.7(6)° (N3-N2-C6-O2) and −176.1(6)° (N2-N3-C8-C9), 178.1(4)° (N1-N2-C15-O1) and 177.8(4)° (N2-N1-C13-C7), 177.4(6)° (N2-N1-C10-O1) and −176.3(6)° (N1-N2-C12-C13), respectively. The methoxyl groups in **I** and **II** are almost co-planar with the benzene ring. The torsion angles of C1-O1-C8-C7, C2-O2-C3-C4 and C15-O3-C12-C11 are found to be 5.0(5)°, −2.7(5)°, and 1.1(11)°, respectively, which are consistent with the literature value of 174.9(4)° [15]. Rings A, B and thiazolidine-2-thione ring D in **II** are twisted 64.12°, 38.34°, and 79.22°, respectively, from the plane of the pyrazole ring, whereas the corresponding angles in **III** are 45.00°, 50.49°, and 69.94°. Rings D in **II** and **III** both adopt envelope conformation, with the C1 and C19 atoms displaced by 0.240 Å and 0.368 Å from the plane of the other ring atoms. The torsion angle of C4-N1-C3-S2 in **II** is −1.0(10)°, whereas the corresponding torsion angle in **III** is 9.4(7)° (C17-N3-C18-S1). Rings A and B in **IV** are twisted 59.92° and 43.65°, respectively, from the plane of the pyrazole ring. An ORTEP view of **IV** reveals that the saccharide moiety in **IV** is a glucopyranose ring in the usual ^4^*C*_1_ conformation. The anomeric center of the saccharide has the β configuration, which is also confirmed from the torsion angles of O1-C19-O2-C20 −173.8(4)° and O2-C19-C23-O10 −174.2(4)°. The torsion angle C21-C20-C26-O4 of −61.0(6)° indicates the acetyl group attached to the primary hydroxyl group is in the *gt* position, which is known to be the favored orientation for a glucopyranose. The pyrazole ring is nearly coplanar with the anomeric C19 atom by making a torsion angle of C19-O1-C10-C11 160.7(6)°, and this orientation facilitates the delocalization of electrons from the lone-pair orbitals of O1 with the π orbitals of the pyrazole ring.

**Figure 2:**
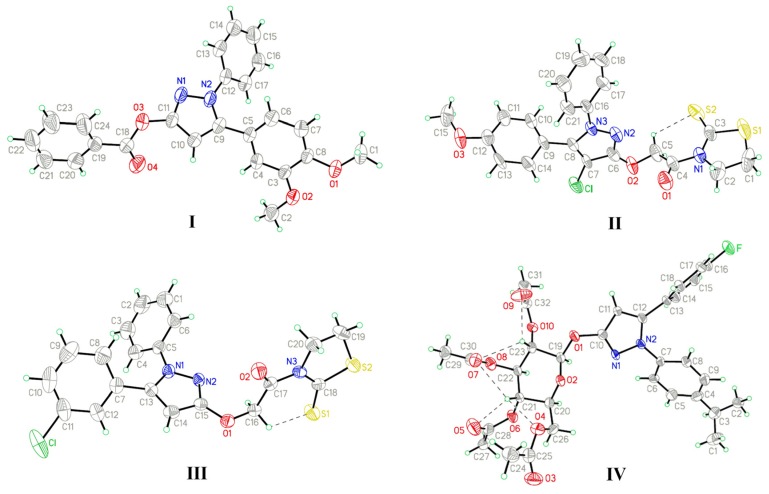
X-ray crystal structures of **I**, **II**, **III**, and **IV**.

**Figure 3:**
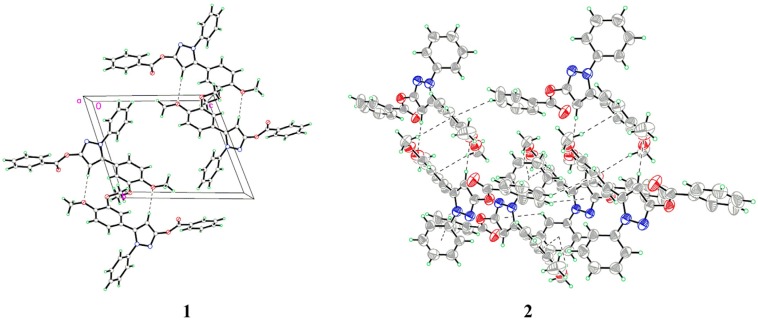
(**1**) A packing diagram of **I**; (**2**) Intermolecular C-H···π interactions of **I**.

**Figure 4:**
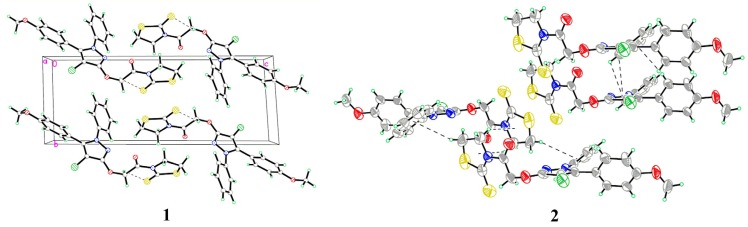
(**1**) A packing diagram of **II**; (**2**) Intermolecular C-H···π interactions of **II**.

**Figure 5:**
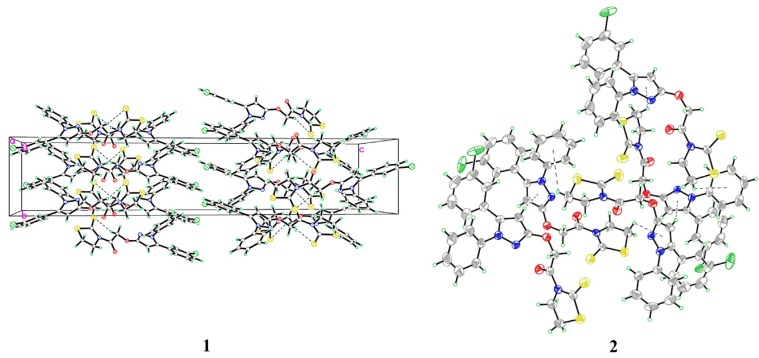
(**1**) A packing diagram of **III**; (**2**) Intermolecular C-H···π interactions of **III**.

**Figure 6:**
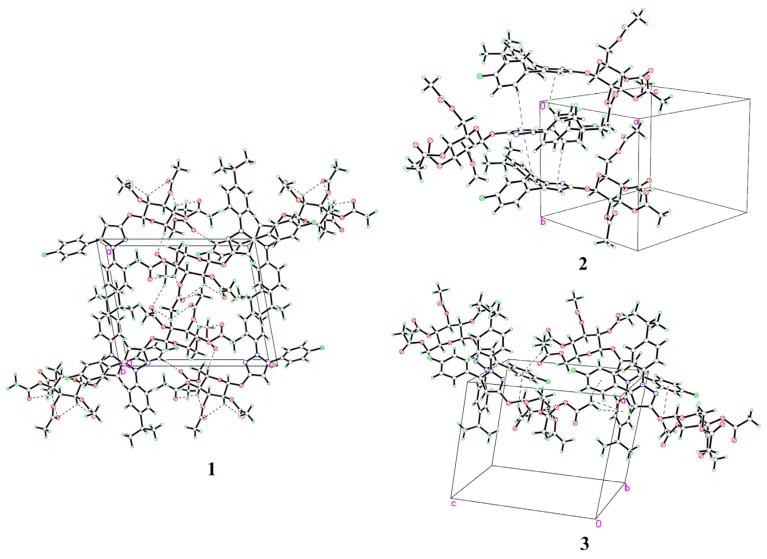
(**1**) A packing diagram of **IV**; (**2**) Intermolecular C-H···π _(pyrazole)_ interactions of **IV**; (**3**) Intermolecular C-H···π _(phenyl)_ interactions of **IV**.

The atom numbering schemes and arrangements for **I-IV** are shown in Figure 2. The molecules are organized in the crystal lattice by classical intramolecular (C-H···S for **II** and **III**, and C-H···O for **IV**) and intermolecular (C-H···O for **I** and **IV**) H-bonds (Table 3a), as well as intermolecular C-H···π stacking interactions (Table 3b). For **I** (Figure 3), there is an intermolecular C-H···O H-bond, between the pyrazole hydrogen and the oxygen atom of the methoxyl group, as well as six C-H···π interactions, between the ring B hydrogen (H17A) and the center of the pyrazole ring (C17-H17A···*Cg*1), the methoxyl hydrogen (H2B), ring A hydrogen (H4A), or phenyl hydrogen (H24A) and the center of the ring B (C2-H2B···*Cg*2, C4-H4A···*Cg*2, and C24-H24A···*Cg*2), as well as the phenyl hydrogen (H22A) or the methoxyl hydrogen (H2C) to the center of the ring A (C22-H22A···*Cg*3 and C2-H2C···*Cg*3), to form a three-dimensional network. For **II** (Figure 4), the intramolecular C5-H5A···S2 H-bond results in the formation of one non-planar pseudo ring (C5/H5A/S2/C3/N1/C4), with H5A atom displaced by 0.580 Å from the plane of the other ring atoms. Five C-H···π interactions, between the methylene hydrogen (H2C) and the center of the ring D (C2-H2C···*Cg*4), the methylene hydrogen (H1A) or the phenyl hydrogen (H14A) and the center of the ring B (C1-H1A···*Cg*2 and C14-H14A···*Cg*2), and the phenyl hydrogens (H17A and H18A) and the center of the pyrazole ring (C17-H17A···*Cg*1 and C18-H18A···*Cg*1), form dimers. For **III** (Figure 5), the structure is stabilized by the intramolecular C-H···S H-bond, between the methylene hydrogen and the sulfur atom of the ring D to form a non-planar pseudo ring (H16B/C16/C17/N3/C18/S1) bearing envelope conformation, with H16B atom displaced by 0.604 Å from the plane of the other atoms, and three C-H···π interactions, one is between the methylene hydrogen (H19B) and the center of the ring B (C19-H19B···*Cg*2), and the others are from the pyrazole hydrogen (H14A) or the methylene hydrogen (H19C) to the center of the pyrazole ring (C14-H14A···*Cg*1 and C19-H19C···*Cg*1). For **IV** (Figure 6), five intra- and four intermolecular H-bonds, and six C-H···π interactions, between the phenyl hydrogens (H14A and H18A) to the center of the pyrazole ring (C14-H14A···*Cg*1 and C18-H18A···*Cg*1), and the methyl hydrogens (H27A-C and H31C) to the centers of the rings A or B (C27-H27A···*Cg*2, C27-H27C···*Cg*2, C27-H27B···*Cg*3, and C31-H31C···*Cg*3), reinforce the crystal packing.

**Table 3:** Parameters (Å, °) for the intra- and intermolecular interactions in **I**, **II**, **III**, and **IV**.

Comp.	D-H…A	D-H	H···A	D···A	D-H···A
*(a) Intermolecular and intramolecular hydrogen bond*					
**I**	C10-H10A···O1 ^a^	0.9300	2.5100	3.442(5)	175.00
**II**	C5-H5A···S2	0.9700	2.5900	3.033(8)	108.00
**III**	C16-H16B···S1	0.9700	2.5800	3.094(5)	113.00
**IV**	C21-H21A···O4	0.9800	2.5300	2.897(8)	102.00
	C21-H21A···O5	0.9800	2.2700	2.645(8)	102.00
	C21-H21A···O7	0.9800	2.4000	2.975(8)	117.00
	C23-H23A···O7	0.9800	2.4000	2.958(7)	116.00
	C23-H23A···O9	0.9800	2.2600	2.659(9)	103.00
	C17-H17A···O3 ^b^	0.9300	2.5200	3.440(10)	170.00
	C20-H20A···O3 ^c^	0.9800	2.4100	3.363(9)	163.00
	C24-H24B···O9 ^d^	0.9600	2.5100	3.375(11)	150.00
	C29-H29C···O7 ^e^	0.9600	2.4200	3.320(10)	155.00
**Comp.**	**C-H···*Cg***	**C-H**	**H···*Cg***	**C···*Cg***	**C-H···*Cg***
*(b) C-H…π interactions*					
**I**	C17-H17A···*Cg*1 ^f^	0.9300	3.2873	3.900(4)	125.39
	C2-H2B···*Cg*2 ^f^	0.9600	3.1975	4.050(5)	148.87
	C4-H4A···*Cg*2 ^f^	0.9300	3.3208	4.113(4)	144.40
	C24-H24A···*Cg*2 ^g^	0.9300	3.3983	4.143(5)	138.64
	C2-H2C···*Cg*3 ^a^	0.9600	2.9016	3.706(5)	142.02
	C22-H22A···*Cg*3 ^h^	0.9300	3.1317	3.866(6)	137.15
**II**	C2-H2C···*Cg*4 ^i^	0.9700	3.0259	3.812(8)	139.03
	C1-H1A···*Cg*2 ^i^	0.9700	3.2864	3.927(8)	125.25
	C14-H14A···*Cg*2 ^j^	0.9300	3.0647	3.906(7)	151.31
	C17-H17A···*Cg*1 ^k^	0.9300	3.2738	3.550(7)	99.63
	C18-H18A···*Cg*1 ^k^	0.9300	3.0448	3.418(9)	105.87
**III**	C19-H19B···*Cg*2 ^l^	0.9700	3.3425	4.032(6)	129.74
	C14-H14A···*Cg*1 ^m^	0.9300	2.8623	3.725(6)	154.68
	C19-H19C···*Cg*1 ^n^	0.9700	3.3978	3.940(6)	117.46
**IV**	C14-H14A···*Cg*1 ^o^	0.9300	3.2004	3.780(9)	122.32
	C18-H18A···*Cg*1 ^p^	0.9300	3.3184	3.865(7)	119.69
	C27-H27A···*Cg*2 ^c^	0.9600	3.0282	3.571(9)	117.24
	C27-H27C···*Cg*2 ^c^	0.9600	3.2317	3.571(9)	102.94
	C27-H27B···*Cg*3 ^q^	0.9600	2.7786	3.647(9)	150.82
	C31-H31C···*Cg*3 ^o^	0.9600	3.3687	4.192(9)	144.97

Symmetry codes: ^a^ 1-x, 2-y, −z; ^b^ x, y, −1+z; ^c^ 1-x, 1/2+y, 1-z; ^d^ 2-x, −1/2+y, 1-z; ^e^ 2-x, 1/2+y, 1-z; ^f^ 1-x, 1-y, −z; ^g^ 2-x, 1-y, −z; ^h^ 1+x, y, −1+z; ^i^ 1-x, -y, 1-z; ^j^ 1+x, y, z; ^k^ −1+x, y, z; ^l^ 2-x, 1/2+y, 1/2-z; ^m^ 3/2-x, 1/2+y, z; ^n^ 2-x, −1/2+y, 1/2-z; ^o^ 1-x, 1/2+y, −z; ^p^ 1-x, −1/2+y, −z; ^q^ x, y, 1+z; Cg1, Cg2, Cg3, and Cg4 are the centroids of the pyrazole ring, *N*-linked benzene ring B, *C*-linked benzene ring A, and thiazolidine-2-thione ring D, respectively.

### 2.2. Experimental and Theoretical FTIR Results

**Figure 7:**
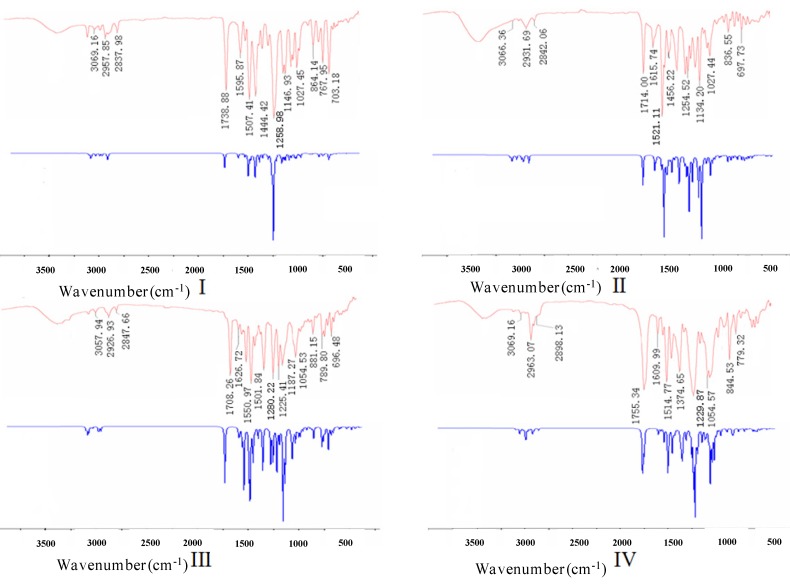
Experimental (above) and theoretical (below) FTIR spectra for **I**, **II**, **III**, and **IV**.

The experimental and theoretical FTIR spectra for **I**–**IV** are shown in Figure 7, where the intensity is plotted against the vibrational frequencies. The primary vibrational frequencies with assignments are listed in Table 4. The main signals are grouped in three regions, including the ranges of 2800–3200 cm^−1^, 1000–1800 cm^−1^ and 500–1000 cm^−1^. The second region shows strongly mixed vibrational bands. The C=O stretching vibrations of **I**–**IV** were found at 1739 cm^−1^, 1714 cm^−1^, 1708 cm^−1^, and 1755 cm^−1^, whereas they were calculated at 1737 cm^−1^, 1718 cm^−1^, 1711 cm^−1^, and 1752 cm^−1^, respectively. The experimental and theoretical C=C stretching vibrations of the phenyl rings were found in the region of 1440–1630 cm^−1^, which were consistent with the literature value of 1,430–1,625 cm^−1^ [16]. The bands observed in the region of 1000–1300 cm^−1^ corresponded to the symmetric and asymmetric C-O stretching vibrations. The region below 1000 cm^−1^ exhibited the out of plane bending C-H vibrations of the aromatic rings, and the region in 3000–3200 cm^−1^ was the characteristic absorption of the aromatic C-H stretching vibrations. The C-Cl stretching vibration in **II** was consistent with the aromatic C-Cl stretching vibration in **III**. The experimental C-F stretching vibration at 1330 cm^−1 in **IV** was consistent with its theoretical value of 1332 cm−1^. These results indicated that the observed and calculated FTIR data were in good agreement with each other.

**Table 4:** Primary vibrations of experimental and theoretical FTIR for **I**, **II**, **III**, and **IV** (cm^−1^).

Vibration	I		II		III		IV	
	Exp.	B3LYP/6-31G *	Exp.	B3LYP/6-31G *	Exp.	B3LYP/6-31G *	Exp.	B3LYP/6-31G *
*ν*_=CH_	3069	3090	3066	3076	3058	3073	3069	3068
*ν*_C-H_	2958	2953	2932	2935	2927	2936	2963	2958
	2838	2842	2842	2847	2848	2845	2898	2896
*ν*_C=O_	1739	1737	1714	1718	1708	1711	1755	1752
*ν*_C=C_	1596	1593	1616	1624	1627	1623	1610	1603
	1549	1548	1521	1521	1595	1604	1556	1555
	1507	1509	1456	1458	1551	1552	1515	1514
	1444	1440			1502	1508	1466	1463
					1466	1459		
*ν*_C-O_	1259	1264	1280	1267	1280	1266	1230	1234
	1167	1168	1255	1267	1225	1240	1161	1163
	1147	1130	1226	1222	1187	1183	1080	1081
	1088	1095	1179	1185	1054	1053	1055	1054
	1066	1071	1134	1147				
	1027	1020	1027	1024				
*γ*_=C-H_	864	869	837	828	881	888	845	846
	811	807	773	782	790	787	779	770
	768	764	698	695	767	774		
	703	703			696	701		
					672	676		
*ν*_C-Cl_			734	736	734	737		
*ν*_C-F_							1330	1332

### 2.3. Fungicidal Activity

Compounds **I**–**IV** were evaluated for *in vitro* fungicidal activity against *Sclerotinia sclerotiorum* and *Gibberella zeae*, at a dosage of 10 μg/mL. As can be seen in Table 5, for *Sclerotinia sclerotiorum*, Compound **IV** (29%) possessing a *per*-*O*-acetylated glucopyranosyl moiety, displayed better activity than **I** (0%), **II** (21%), and **III** (14%).

**Table 5:** *In vitro* fungicidal activities of **I**, **II**, **III**, and **IV** (% inhibition).

Comp.	X	Y	Inhibition rate (%)	
			*S. sclerotiorum*	*G. zeae*
**I**	3,4-(OCH_3_)_2_	H	0	4
**II**	*p*-OMe	H	21	32
**III**	*m*-Cl	H	14	29
**IV**	*p*-F	*p*-Me_2_CH	29	11

The reason was speculated that the glucopyranosyl moiety could form more H-bonds (Figure 2) to improve the hydrophilicity of molecule, which could balance the HLB value and then increase the systemic of molecule within plant. Within the series of thiazolidinethione derivatives, compound **II** (21%, 32%) with an electron-withdrawing chloro group on the pyrazole ring displayed better fungicidal activity against the two fungi than **III** (14%, 29%), and both compounds showed better inhibitory activity against *Gibberella zeae* than **I** (4%) and **IV** (11%). The results might imply that the introduction of the heterocycle moiety by full consideration of the electronic effects was important for improving its fungicidal activity. However, compound **I** containing a benzoyloxy moiety showed the worst activity (0, 4%), which indicated switching the C3-substituent of the pyrazole ring from thiazolidinethione to benzoyloxy moiety had no effective impact on the inhibition rates. The structure-activity relationship revealed that the improvement of bioactivity might require a reasonable design of molecules (e.g., considering H-bonds effect and heterocycle) and full consideration of the electronic effects of electron-withdrawing groups to balance the HLB value and enhance the systemic of the whole molecule.

## 3. Experimental

### 3.1. General Information

Melting points were measured on an X-4 microscope electrothermal apparatus (Taike, Nanjing, China) and were uncorrected. ^1^H-NMR spectra were recorded on a Bruker spectrometer (Bruker, Leipzig, Germany) at 300 or 500 MHz using CDCl_3_ or DMSO-*d*_6_ as solvent, with tetramethylsilane as an internal standard. Elemental analyses were performed on a Flash EA-1112 elemental analyzer (Thermo, Illinois, USA). FTIR spectra of compounds **I**–**IV** were recorded in the region of 4000–400 cm^−1^ on a Nicolet 380 FT-IR spectrophotometer (Thermo, Waltham, MA, USA) using the KBr pellet technique.

### 3.2. Synthesis and Characterization

Compounds **I**–**IV** were prepared from 1,5-diaryl-1*H*-pyrazol-3-ols *via* a series of reactions that included substitution, hydrolysis, condensation, chlorination, glycosylation, or esterification [8,9,10,11,12].

#### 3.2.1. Preparation of Compound **I**

To a solution of 5-(3,4-dimethoxyphenyl)-1-phenyl-1*H*-pyrazol-3-ol (0.59 g, 2.0 mmol) in CHCl_3_ (50 mL) was added Et_3_N (0.41 g, 4.0 mmol). The mixture was stirred for 10 min and benzoyl chloride (0.42 g, 3.0 mmol) was added. Then, the mixture was stirred at r.t. for 2 h. The solvent was removed under reduced pressure and the residue was recrystallized from ethanol to give the white solid product 5-(3,4-dimethoxyphenyl)-1-phenyl-3-benzoyloxy-1*H*-pyrazole (**I**). Yield: 87%; M.p. 133–134 °C; ^1^H-NMR (300 MHz, DMSO-*d_6_*) *δ*: 8.16 (d, *J* = 7.2 Hz, 2H, Ar-H), 7.79 (t, *J* = 7.3 Hz, 1H, Ar-H), 7.65 (t, *J* = 7.3 Hz, 2H, Ar-H), 7.48–7.31 (m, 5H, Ar-H), 7.00–6.80 (m, 3H, Ar-H), 6.67 (s, 1H, CH), 3.76 (s, 3H, OCH_3_), 3.57 (s, 3H, OCH_3_); Anal. Calcd for C_24_H_20_N_2_O_4_: C 71.99, H 5.03, N 7.00; found C 71.75, H 5.01, N 7.03.

#### 3.2.2. Preparation of Compound **II**

SOCl_2_ (5 mL) was put into a round-bottom flask and then 2-((5-(4-methoxyphenyl)-1-phenyl-1*H*-pyrazol-3-yl)oxy)-1-(2-thioxothiazolidin-3-yl)ethanone (0.43 g, 1.0 mmol) as well as catalytic amount of DMF (0.1 mmol) was added. The mixture was heated under reflux for 4 h. Then excess SOCl_2_ was evaporated under reduced pressure and H_2_O (200 mL) was added with good stirring. The precipitate was filtered off, washed with water and dried. It was then purified by flash column chromatography (eluent: ethyl acetate/petroleum ether, 1:6 *v*/*v*) to afford the yellow solid product 2-((4-chloro-5-(4-methoxyphenyl)-1-phenyl-1*H*-pyrazol-3-yl)oxy)-1-(2-thioxothiazolid-in-3-yl)ethanone (**II**). Yield: 65%; M.p. 193–194 °C; ^1^H-NMR (500 MHz, CDCl_3_) *δ*: 7.26–7.15 (m, 7H, Ar-H), 6.88 (d, *J* = 8.8 Hz, 2H, Ar-H), 5.78 (s, 2H, CH_2_), 4.61 (t, *J* = 7.6 Hz, 2H, CH_2_), 3.82 (s, 3H, OCH_3_), 3.38 (t, *J* = 7.6 Hz, 2H, CH_2_); Anal. Calcd for C_21_H_18_ClN_3_O_3_S_2_: C 54.84, H 3.94, N 9.14; found C 54.95, H 3.95, N 9.17.

#### 3.2.3. Preparation of Compound **III**

2-((5-(3-Chlorophenyl)-1-phenyl-1*H*-pyrazol-3-yl)oxy)acetic acid (0.33 g, 1.0 mmol) was dissolved in a solution of DCC (0.22 g, 1.05 mmol) in CH_2_Cl_2_ (50 mL), and the mixture was stirred at 0 °C for 1 h. Then, thiazolidine-2-thione (0.12 g, 1.0 mmol) and DMAP (0.01 g, 0.1 mmol) was added. The solution was stirred at 0 °C for 2 h and then at room temperature for 12 h. The white precipitate was filtered off and the solvent was evaporated under reduced pressure. The residue was purified by flash column chromatography over silica gel eluting with petroleum ether/ethyl acetate 3:1 to gain the the yellow solid product 2-((5-(3-chlorophenyl)-1-phenyl-1*H*-pyrazol-3-yl)oxy)-1-(2-thioxothiazolidin-3-yl)ethanone (**III**). Yield: 73%; M.p. 143–144 °C; ^1^H-NMR (500 MHz, CDCl_3_) *δ*: 7.30–7.05 (m, 9H, Ar-H), 6.05 (s, 1H, CH), 5.72 (s, 2H, CH_2_), 4.60 (t, *J* = 7.6 Hz, 2H, CH_2_), 3.37 (t, *J* = 7.6 Hz, 2H, CH_2_); Anal. Calcd for C_20_H_16_ClN_3_O_2_S_2_: C 55.87, H 3.75, N 9.77; found C 55.78, H 3.74, N 9.80.

#### 3.2.4. Preparation of Compound **IV**

A mixture of CHCl_3_ (20 mL), Bu_4_N^+^Br^−^ (0.23 g, 0.7 mmol), and H_2_O (2 mL) was heated to 55 °C, and then a solution of 1-(4-fluorophenyl)-5-(4-isopropylphenyl)-1*H*-pyrazol-3-ol (0.30 g, 1.0 mmol) in CHCl_3_ (15 mL) and 5% aqueous NaOH (6 mL) were added dropwise. The pH value was adjusted to 8–10, and acetobromo-*α*-D-glucose (0.54 g, 1.3 mmol) was added under vigorously stirring. The mixture was stirred at 55 °C for another 4 h, and then left to cool to room temperature. The organic layer was separated, washed with 5% aqueous NaOH, and dried. Then, the solvent was removed *in vacuo* and the residue was recrystallized from ethanol to give the white solid product 3-(2′,3′,4′,6′-tetra-*O*-acetyl-*β*-D-glucopyranosyloxy)-1-(4-isopropylphenyl)-5-(4-fluorophenyl)-1*H*-pyrazole (**IV**). Yield: 46%; M.p. 151–152 °C. ^1^H-NMR (500 MHz, CDCl_3_) *δ*: 7.26–6.97 (m, 8H, Ar-H), 5.99 (s, 1H, CH), 5.67 (d, *J* = 7.7 Hz, 1H, H_1′_), 5.32–5.26 (m, 2H, H_2′_, H_3′_), 5.19 (t, *J* = 9.5 Hz, 1H, H_4′_), 4.28 (dd, *J* = 4.7, 12.4 Hz, 1H, H_6′b_), 4.18 (dd, *J* = 2.4, 12.4 Hz, 1H, H_6′a_), 3.91–3.88 (m, 1H, H_5′_), 2.93–2.87 (m, 1H, CH), 2.05, 2.04, 2.03, 2.01 (4× s, 12H, 4× COCH_3_), 1.23 (d, *J* = 6.9 Hz, 6H, CH_3_); Anal. Calcd for C_32_H_35_FN_2_O_10_: C 61.34, H 5.63, N 4.47; found C 61.52, H 5.61, N 4.45.

### 3.3. X-ray Crystallography

Suitable crystals of **I**–**IV** were obtained by slow evaporation of ethyl acetate solutions at r.t. Crystal data were performed on a Nonius CAD-4 diffractometer (Enraf-Nonius, Rotterdam, The Netherlands) by using Mo*K*_α_ (λ = 0.71073 Å) irradiation. All of the structures were solved by direct methods using SHELXS-97 and refined by full-matrix least-squares on *F*^2^ for all data using SHELXL-97 [17]. All non-H-atoms were refined anisotropically, and H-atoms were introduced at calculated positions. The isotropic temperature factors were fixed to 1.2 times (1.5 times for methyl group) the equivalent isotropic displacement parameters of the C-atom the H-atom is attached to CCDC-906894 (**I**), CCDC-819778 (**II**), CCDC-918082 (**III**) and CCDC-925316 (**IV**) contain the supplementary crystallographic data for this paper. These data can be obtained free of charge via http://www.ccdc.cam.ac.uk/conts/retrieving.html (or from the CCDC, 12 Union Road, Cambridge CB2 1EZ, UK; Fax: +44 1223 336033; E-mail: deposit@ccdc.cam.ac.uk).

### 3.4. FTIR Spectra

The structures in the ground state (*in vacuo*) were optimized by the Gaussian 03 program using the B3LYP (DFT) method with the 6-31G (d) basis set [18,19,20]. The initial configurations for calculation were constructed according to the X-ray data. Frequency calculations at the same levels of theory revealed no imaginary frequencies, indicating that the B3LYP/6-31G (d) method was the optimal one in our system.

### 3.5. Fungicidal Activity Assays

The *in vitro* fungicidal activity of compounds **I**–**IV** against *Sclerotinia sclerotiorum* and *Gibberella zeae* was investigated at a dosage of 10 μg/mL, according to a reported method [10]. The fungi were obtained from Jiangsu Pesticide Research Institute Co., Ltd., Nanjing, China. The tested compounds **I**–**IV** were dissolved in acetone and added to a sterile agarized Czapek*-*Dox medium at 45 °C. In preliminary screenings, the compounds were used in a concentration of 10 μg/mL. The control sample contained only one equivalent of acetone. The media were poured onto 8-cm Petri dishes (10 mL for each dish) and after 2 days inoculated with 5-mm PDA discs of overgrown mycelium. In the case of *Sclerotinia sclerotiorum*, the medium was inoculated by a prick of laboratory needle containing fungus spores. The *Petri* dishes were incubated at r.t. in the dark. After 4 days, the diameters of the inoculation of the cultures were measured. The percentage inhibition of fungal growth was determined by comparison between the development of fungi colonies on media containing compounds and on the control. Three replicates of each test were carried out.

## 4. Conclusions

Four 1,5-diaryl-3-oxypyrazoles containing benzoyl (**I**), thiazolidinethione (**II** and **III**) or *per*-*O*-acetylated glucopyranosyl (**IV**) moieties have been analyzed by X-ray diffraction. The molecules were stabilized by classical intra- and intermolecular H-bonds, as well as intermolecular C-H···π stacking interactions. Compound **IV** with more H-bonds in the crystal displayed better activity (29%) against *Sclerotinia sclerotiorum* than **I** (0%), **II** (21%), and **III** (14%). Compound **II** (21%, 32%) showed better fungicidal activity against the two fungi than **III** (14%, 29%), and both **II** and **III** exhibited better inhibitory activity against *Gibberella zeae* than **I** (4%) and **IV** (11%). The structure-activity relationship revealed that the improvement of bioactivity might require full consideration of H-bonds effect, heterocycle, and electronic effects of electron-withdrawing groups to balance the HLB value and enhance the systemic of molecule.
